# Model-Based Control of a Continuum Manipulator with Online Jacobian Error Compensation Using Kalman Filtering

**DOI:** 10.34133/cbsystems.0339

**Published:** 2025-08-07

**Authors:** Yujia Zhai, Jihao Xu, Hangjie Mo, Chunqi Zhang, Dong Sun

**Affiliations:** ^1^ Department of Biomedical Engineering, City University of Hong Kong, Hong Kong SAR, China.; ^2^ School of Management, Hefei University of Technology, Hefei 230009, China.

## Abstract

Flexible continuum robots exhibit excellent adaptability to a wide range of tasks and environments. However, accurate and efficient modeling and control remain challenging due to their inherent nonlinearities. In this article, a hybrid model-based and online data-driven control method is proposed for a tendon-driven continuum robot, which requires no prior dataset collection or training. The method incorporates the Jacobian derived from the piecewise constant curvature model with online Jacobian error compensation using a Kalman filter. Consecutive Jacobian estimates are constrained to reduce fluctuations and improve stability in real-time estimation. Experimental results validate the effectiveness of the proposed hybrid approach in enhancing tracking accuracy and demonstrate its robustness against external disturbances.

## Introduction

Continuum robots have recently attracted considerable attention as a promising alternative to traditional rigid-link manipulators. Inspired by natural appendages like elephant trunks, these robots exhibit inherent compliance with infinite degrees of freedom and the ability to adapt seamlessly to various objects and environments [1]. Their flexibility and dexterity have enabled their widespread applications in fields such as robotic surgery [2–4], navigation and inspection in confined spaces [5–7], and collaborative manipulation tasks [8–11]. Nevertheless, the large deformations, frictional effects, and intrinsic nonlinearities of continuum robots pose substantial challenges for precise modeling and control [12,13].

The modeling of continuum robots has been extensively explored using various principle-based approaches rooted in kinematics and mechanics. The pseudo-rigid body theory approximates the continuous shape of the robot as a redundant rigid-link manipulator [14]. The piecewise constant curvature (PCC) model represents each continuum segment as a circular arc [15], offering computational efficiency but potentially compromising precision due to its simplistic assumptions. In Ref. [16], an enhanced state parameterization was proposed to address the singularities inherent in the PCC model. To capture higher-order nonlinearities, variable curvature models were developed by representing the backbone shape with Euler arc splines [17] or by parameterizing the curvature with polynomials [18,19]. From a mechanics-based perspective, Cosserat rod theory characterizes these robots through differential equations that account for forces and torques distributed along the robot’s body [20,21], making it effective for analyzing the influence of tendon routings and parallel structures in continuum robots. The finite element method (FEM), a more general framework for modeling deformable objects, is also applied to study the behavior of continuum robots [22,23]. While both the Cosserat rod model and FEM achieve high accuracy, they come with substantial computational costs.

Alternatively, data-driven methods have been widely employed to model and control continuum robots based on input–output data, without considering the complex physical relationships. For instance, neural networks have been leveraged to directly learn the inverse kinematics of continuum robots [4,8,10,24]. In Refs. [25,26], Koopman operator theory was applied to model the dynamics of soft continuum robots in data-driven frameworks alongside optimal control methods. Without requiring prior training, Lu et al. [27] proposed an online learning approach that adaptively controls continuum robots using radial basis function (RBF) networks. Alambeigi et al. [28,29] presented real-time Jacobian calculation for surgical continuum manipulators using Broyden’s method. Mo et al. [30] improved the robustness of online Jacobian estimation for a continuum manipulator in an endoscopic system by incorporating a sensing data buffer. Li et al. [31] proposed an adaptive Kalman filter to directly estimate the Jacobian of a pneumatic continuum robot in real time. Furthermore, in Ref. [32], a combined offline and online framework was introduced to control an industrial continuum manipulator by integrating a neural network with Broyden’s update rule. In Ref. [33], localized Gaussian process regression was employed to control a continuum robot incorporating online dataset updating.

Additionally, researchers have explored hybrid model-based and data-driven methods for continuum robots, combining the advantages of both manners. Huang et al. [34] integrated physical models with neural networks to account for the unmodeled nonlinear effects of a parallel continuum robot. Li et al. [35] utilized the RBF network to interpolate the Jacobian of a continuum manipulator globally based on a Jacobian set calculated from the Cosserat model. In Refs. [36,37], data-driven models were employed as solution encoders to enhance computational efficiency when solving principle-based models of continuum robots. In Ref. [38], a hybrid Jacobian estimation strategy was proposed for concentric tube robots, using the model-based result as a weighted initial estimate. To improve modeling accuracy, data-driven approaches were employed in Refs. [39,40] to learn the residual kinematics of continuum robots.

**Fig. 1.**
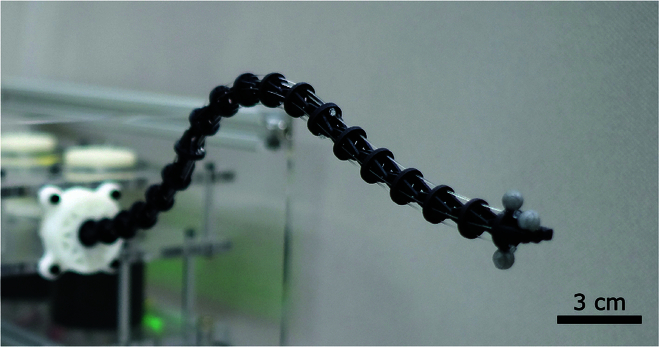
Prototype of the tendon-driven continuum robot. Three reflective markers are attached to the end-effector for visual positioning.

In this article, we propose a hybrid model-based and data-driven control framework for a continuum robot, where the model-based Jacobian error is estimated and compensated online with a Kalman filter. The kinematics of the continuum robot are initially established based on the PCC model. To address the modeling inaccuracies of the PCC model, a Kalman filter is employed to estimate the Jacobian error online, using real-time actuation inputs and end-effector pose measurements. Offline data collection or training is not required with the proposed method. For experimental validation, we design and fabricate a 3-segment tendon-driven continuum manipulator as illustrated in Fig. 1, with the end-effector pose measurements obtained via visual feedback. The effectiveness of the proposed method is demonstrated through a series of trajectory tracking experiments. Comparative experiments are conducted to evaluate the influence of various model parameters. Furthermore, the robustness of the proposed method under external disturbances is validated by applying unexpected payloads to the continuum robot.

This study assumes that the continuum robot operates in a quasi-static manner, with small movement between consecutive time steps. This assumption allows the transition and measurement models of the Jacobian error to be approximated linearly with additive stochastic noise. Consequently, the Jacobian error can be efficiently estimated using a linear Kalman filter. To mitigate numerical oscillations in the Jacobian elements during real-time estimation, additional constraints are applied to the Jacobian estimate. The robot’s Jacobian is divided into 2 components: the position Jacobian and the attitude Jacobian. Thresholds are defined for each component to limit their variation between consecutive iterations. This approach enhances the robustness of the online estimation against noise and disturbances, resulting in smooth Jacobian estimates and, consequently, smooth robot trajectories in closed-loop control.

Compared to mechanics-based models such as Cosserat rod theory [20,21] and FEM [22,23], which are computationally costly, the proposed method can be computed efficiently by integrating the PCC model with a Kalman filter. Additionally, the prediction precision of the PCC model, which is generally limited, is enhanced within the proposed method using real-time measurements. Meanwhile, for online Jacobian estimation, rather than relying solely on measurements, the proposed method leverages the prediction of the kinematic model, enabling fast convergence [28,29,38]. In contrast to learning-based approaches such as neural networks [4,8,10,24] and data-driven Koopman operator theory [25,26], which require large datasets for training, the proposed method eliminates the need for offline data collection or training, making it convenient to implement.

## Materials and Methods

This section first presents the mechanical design of the tendon-driven continuum manipulator. Subsequently, the kinematic modeling and closed-loop control of the developed continuum robot are elaborated, incorporating an online Jacobian error compensation method to enhance control performance. For the simplification of modeling and control, we assume that the movement of the continuum robot is quasi-static and neglect its dynamics.

### Continuum manipulator design

**Fig. 2.**
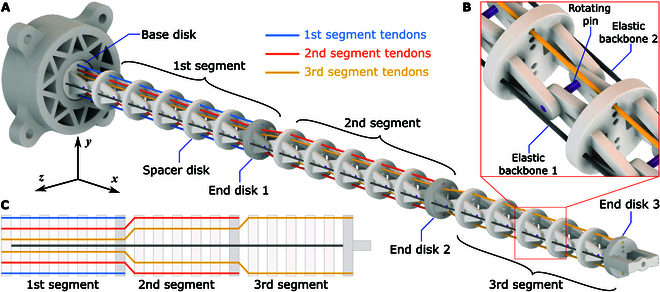
Design of the continuum manipulator. (A) 3D model of the manipulator structure, consisting of 3 continuum segments. The manipulator operates in the x−y plane. (B) Close-up view of the revolute joint between adjacent disks. (C) Diagram illustrating the tendon routing inside the manipulator.

The continuum manipulator is composed of 3 segments, as shown in Fig. 2. Each segment features an end disk for tendon attachment and 5 spacer disks for tendon guidance. The manipulator is mounted to the base via an additional base disk. All disks are connected to the preceding disk through a passive revolute joint with a rotating pin, as illustrated in Fig. 2B. This mechanical design constrains the manipulator’s bending motion to the x−y plane. Additionally, 2 elastic backbones, passing through all the disks, are attached to the end-effector and the base disk, providing the robot with the required stiffness and flexibility.

In Fig. 2, the tendons of different segments are marked with distinct colors for clear identification. Each continuum segment is actuated by 2 tendons in an antagonistic manner. As shown in Fig. 2C, the driving tendons are attached only to the end disk of the respective segment and slide through all preceding disks via specifically designed holes. The offsets of the tendon pairs from the disk center are adjusted along the backbone to avoid interaction and friction between tendons. Each tendon, after passing through the base disk, is independently connected to a motor via a capstan. The configuration of the continuum manipulator is controlled by adjusting the lengths of the 6 tendons using 6 motors.

### PCC model

**Fig. 3.**
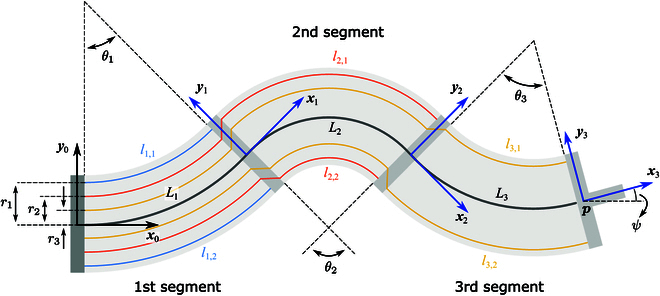
Diagram depicting the modeling of the 3-segment continuum robot. The inertial frame $\left\{x_{0},\;y_{0}\right\}$ is centered at the base disk, while the body-fixed frame for each segment $\left\{x_{i},\;y_{i}\right\}$ $\left(i=1,\;2,\;3\right)$ is assigned to the center of its respective end disk.

To simplify the kinematics, the shape of each segment of the continuum robot is assumed as a circular arc, as shown in Fig. 3. The inertial frame $\left\{x_{0},\;y_{0}\right\}$ is defined at the center of the base disk, while the body-fixed frame $\left\{x_{i},\;y_{i}\right\}$ $\left(i=1,\;2,\;3\right)$ is located at the center of the end disk of the *i*th segment. The *x* axes of all coordinate frames are aligned with the corresponding tangent directions of the backbone. For the *i*th segment, the backbone length and the bending angle are denoted by $L_{i}$ and $\theta_{i}$, respectively, while the lengths of the 2 driving tendons are represented by $l_{i,1}$ and $l_{i,2}$, as depicted in Fig. 3.

In accordance with the geometrical relationships, the homogeneous transformation from frame $\left\{x_{i},\;y_{i}\right\}$ to frame $\left\{x_{i-1},\;y_{i-1}\right\}$ can be expressed as

$$T_{i-1}^{i}=\left[\begin{array}{ccc} \text{cos}\theta_{i} & -\text{sin}\theta_{i} & \frac{L_{i}}{\theta_{i}}\text{sin}\theta_{i}\\ \text{sin}\theta_{i} & \text{cos}\theta_{i} & \frac{L_{i}}{\theta_{i}}\left(1-\text{cos}\theta_{i}\right)\\ 0 & 0 & 1 \end{array}\right]$$ (1)

Thus, the homogeneous transformation from the robot’s end-effector $\left\{x_{3},\;y_{3}\right\}$ to the base disk $\left\{x_{0},\;y_{0}\right\}$ is obtained as

$$T_{0}^{3}=T_{0}^{1}T_{1}^{2}T_{2}^{3}=\left[\begin{array}{ccc} \text{cos}\psi & -\text{sin}\psi & x\\ \text{sin}\psi & \text{cos}\psi & y\\ 0 & 0 & 1 \end{array}\right]$$ (2)

where $p={\left[x,\;y\right]}^{T}$ is the position of the end-effector with

$$\begin{array}{c} \begin{array}{c} x=\frac{L_{1}}{\theta_{1}}\text{sin}\theta_{1}+\frac{L_{2}}{\theta_{2}}\left(\text{sin}\left(\theta_{1}+\theta_{2}\right)-\text{sin}\theta_{1}\right)\\ \;+\frac{L_{3}}{\theta_{3}}\left(\text{sin}\left(\theta_{1}+\theta_{2}+\theta_{3}\right)-\text{sin}\left(\theta_{1}+\theta_{2}\right)\right) \end{array} \end{array}$$ (3)

$$\begin{array}{c} \begin{array}{l} y=\frac{L_{1}}{\theta_{1}}\left(1-\text{cos}\theta_{1}\right)+\frac{L_{2}}{\theta_{2}}\left(\text{cos}\theta_{1}-\text{cos}\left(\theta_{1}+\theta_{2}\right)\right)\\ \;+\frac{L_{3}}{\theta_{3}}\left(\text{cos}\left(\theta_{1}+\theta_{2}\right)-\text{cos}\left(\theta_{1}+\theta_{2}+\theta_{3}\right)\right) \end{array} \end{array}$$ (4)

and ψ denotes the end-effector’s attitude:

$$\psi =\theta_{1}+\theta_{2}+\theta_{3}$$ (5)

Here, p and ψ are taken as the outputs produced by the continuum robot.

To actuate the continuum manipulator, the lengths of 3 pairs of tendons are regulated. For the 2 driving tendons of the same segment, when one is elongated, the other is assumed to shorten correspondingly by the same amount. Denoting $\Delta l_{i}$ as the tendon length change of the *i*th segment, we have

$$\Delta l_{i}=\sum_{j=1}^{i}L_{j}-l_{i,1}=l_{i,\;2}-\sum_{j=1}^{i}L_{j},\;i=1,\;2,\;3$$ (6)

The actuation inputs of the robot can be expressed in vector form as $u={\left[\Delta l_{1},\;\Delta l_{2},\;\Delta l_{3}\right]}^{T}$. Based on the geometric relationships, the connection between the robot’s bending angles and the actuation inputs is obtained as

$$r_{1}\theta_{1}=\Delta l_{1}$$ (7)

$$r_{2}\theta_{1}+r_{1}\theta_{2}=\Delta l_{2}$$ (8)

$$r_{3}\theta_{1}+r_{2}\theta_{2}+r_{1}\theta_{3}=\Delta l_{3}$$ (9)

where $r_{1}$, $r_{2}$, and $r_{3}$ are the offsets between the tendons and the disk center, as illustrated in Fig. 3. Solving $\theta_{1}$, $\theta_{2}$, and $\theta_{3}$ from Eqs. (7) to (9), and substituting them into Eqs. (3) to (5), establishes a direct relationship between the end-effector pose and the actuation inputs as $p=p\left(u\right)$ and $\psi =\psi \left(u\right)$. Furthermore, by taking the derivative with respect to the actuation inputs, the Jacobian of the robot can be derived as

Jm=JpmJψm=∂p∂u∂ψ∂u∈ℝ3×3 (10)

where the superscript ⋅m denotes the Jacobian calculated directly using the PCC model, Jpm∈ℝ2×3 represents the position Jacobian, and Jψm∈ℝ1×3 represents the attitude Jacobian. Based on the robot’s Jacobian, a closed-loop controller can be conveniently developed, as elaborated later.

### Online Jacobian error compensation

Due to the strong assumption of the piecewise circular shape, the modeling precision of the PCC model is generally limited. Specifically, there exists an error between Jm, the Jacobian of the PCC model given by Eq. (10), and J, the actual Jacobian of the continuum robot. Define δJ=J−Jm, where $\delta J\in {\mathbb{R}}^{3\times 3}$ represents the Jacobian error of the PCC model caused by modeling inaccuracies. To enhance the modeling precision, a Kalman filter is employed to estimate δJ and compensate for the model-based Jacobian in real time.

The Jacobian error is considered as the state of the Kalman filter. Specifically, for ease of implementation, the state vector of the Kalman filter is defined as $\xi =\delta J^{\vee }\in {\mathbb{R}}^{9}$, where the operator ${\left(\cdot \right)}^{\vee }:{\mathbb{R}}^{3\times 3}\mapsto {\mathbb{R}}^{9}$ concatenates a matrix into a column vector by stacking its row vectors. Based on the quasi-static movement assumption of the continuum manipulator, we assume that the evolution of the Jacobian error between adjacent time steps is linear with additive stochastic noise. Therefore, the state transition model is expressed in discrete form as

$$\xi_{k}=\gamma \xi_{k-1}+w_{k-1}$$ (11)

where *k* denotes the time index, $\gamma \in \left[0,\;1\right]$ is a tunable fading rate to determine the influence of the previous state, and $w_{k-1}\in {\mathbb{R}}^{9}$ is Gaussian noise with zero mean and covariance matrix $Q_{k-1}\in {\mathbb{R}}^{9\times 9}$. Correspondingly, the estimated state and its covariance matrix $P_{k}\in {\mathbb{R}}^{9\times 9}$ are propagated as

$$\xi_{k}^{-}=\gamma \xi_{k-1}^{+}$$ (12)

$$P_{k}^{-}=\gamma^{2}P_{k-1}^{+}+Q_{k-1}$$ (13)

where the notations ${\left(\cdot \right)}^{-}$ and ${\left(\cdot \right)}^{+}$ represent the prior and posterior estimates, respectively.

The prior estimate of the Jacobian error is subsequently updated using the actuation inputs and the end-effector pose measurements. Under the assumption of quasi-static motion, the robot movement between consecutive time steps is small. Thus, the measurement of the Jacobian error is approximated through a linear model with additive stochastic noise as

pk−pk−1ψk−ψk−1=HkJk∨m+ξk+vk (14)

where $v_{k}\in {\mathbb{R}}^{3}$ is zero-mean Gaussian noise with covariance matrix $R_{k}\in {\mathbb{R}}^{3\times 3}$, Jk∨m is the vectorized form of the Jacobian of the PCC model, and $H_{k}\in {\mathbb{R}}^{3\times 9}$ is the measurement matrix, given by

$$H_{k}=\left[\begin{array}{ccc} u_{k}^{T}-u_{k-1}^{T} & 0_{1\times 3} & 0_{1\times 3}\\ 0_{1\times 3} & u_{k}^{T}-u_{k-1}^{T} & 0_{1\times 3}\\ 0_{1\times 3} & 0_{1\times 3} & u_{k}^{T}-u_{k-1}^{T} \end{array}\right]$$ (15)

with $0_{1\times 3}$ being a 1×3 all-zero row vector. The covariance matrices $Q_{k-1}$ and $R_{k}$ balance the filter’s reliance on the prior estimation and the measurement [41]. Subsequently, the Kalman gain $K_{k}\in {\mathbb{R}}^{9\times 3}$ is computed as

$$K_{k}=P_{k}^{-}H_{k}^{T}{\left(H_{k}P_{k}^{-}H_{k}^{T}+R_{k}\right)}^{-1}$$ (16)

Then, the posterior estimate of the Jacobian error and its covariance matrix are given by

ξk+=ξk−+Kkpk−pk−1ψk−ψk−1−HkJk∨m+ξk− (17)

$$P_{k}^{+}=\left(I_{9}-K_{k}H_{k}\right)P_{k}^{-}$$ (18)

where $I_{9}$ is a 9×9 identity matrix. Finally, the estimated Jacobian of the continuum robot can be obtained as

Jke=Jkm+δJk+ (19)

where $\delta J_{k}^{+}$ is reconstructed from its vectorized form $\xi_{k}^{+}$ given in Eq. (17) provided by the Kalman filter.

Furthermore, to avoid dramatic variation of the estimated Jacobian during the real-time estimation process, additional constraints are applied to restrict the updates. Denoting Jc as the constrained Jacobian, it is calculated as

Jkc=Jp,kcJψ,kc=Jp,ke,ifJp,ke−Jp,k−1c⩽σpJp,k−1c+σpJp,ke−Jp,k−1cJp,ke−Jp,k−1c,otherwiseJψ,ke,ifJψ,ke−Jψ,k−1c⩽σψJψ,k−1c+σψJψ,ke−Jψ,k−1cJψ,ke−Jψ,k−1c,otherwise (20)

where the operator $\left\|\cdot \right\|$ represents the Frobenius norm, and $\sigma_{p}$ and $\sigma_{\psi }$ are tunable thresholds to limit the variation magnitudes of the position Jacobian and the attitude Jacobian, respectively. The superscripts ⋅e and ⋅c distinguish the unconstrained and constrained Jacobian estimates, respectively.

### Closed-loop control

To control the continuum robot to track the reference pose trajectory ${\left[p_{r}^{T},\;\psi_{r}\right]}^{T}$, a Jacobian-based control law is applied in discrete form as

uk+1=uk+βJk†cprψrk−pψk (21)

where β is a controller gain that can be tuned according to [32], and Jk†c is the damped pseudoinverse of the constrained Jacobian estimate, given by

**Fig. 4.**
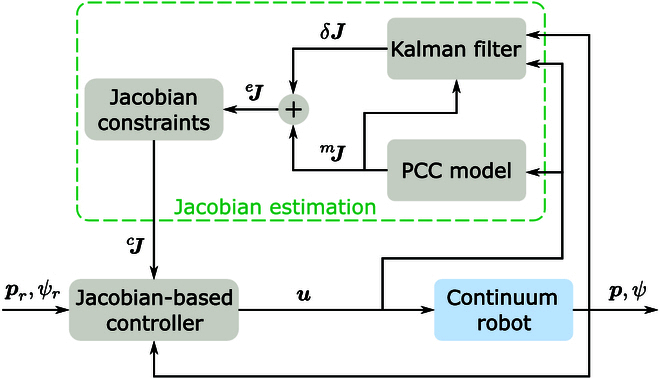
Block diagram of the control scheme with online Jacobian error compensation.

Jk†c=JkTccJk+αI3−1JkTc (22)

where $I_{3}$ is a 3×3 identity matrix, and α is a small positive constant added to prevent singularities when computing the matrix inverse. Fig. 4 illustrates the block diagram of the control strategy. The controller leverages the model-based Jacobian with online compensation, which alleviates modeling errors and enhances control performance.

## Results

To verify the effectiveness of the proposed online Jacobian error compensation method, experiments were conducted on the continuum manipulator platform. The trajectory tracking capabilities, the behaviors under parameter variations, as well as the performance under external disturbances were tested and demonstrated.

### Experimental setup

**Fig. 5.**
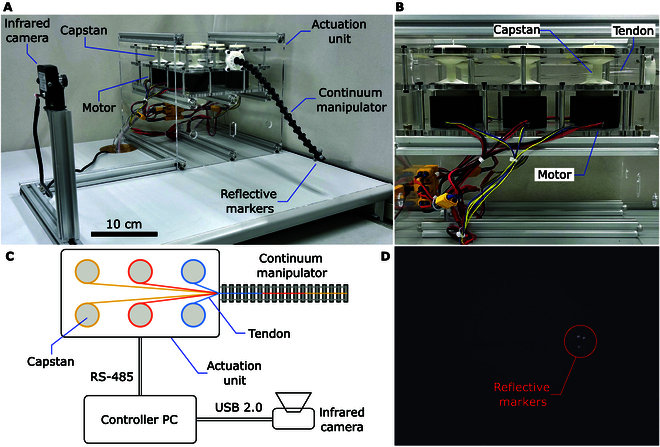
Experimental setup for conducting closed-loop control of the continuum manipulator. (A) Photograph of the platform. (B) Side view of the interior of the actuation unit. (C) Schematic diagram of the platform. (D) Image captured by the infrared camera with 3 reflective markers labeled.

Fig. 5 illustrates the setup of the experimental platform. The closed-loop control of the continuum robot was implemented with 3 primary components: the continuum manipulator as the main robotic structure, the actuation unit for driving the manipulator, and the infrared camera for providing real-time feedback.

The continuum manipulator consists of 3 segments, each comprising 5 spacer disks and 1 end disk, resulting in a diameter of 13 mm and a total length of 270 mm. The disks are 3-dimensionally (3D) printed from nylon and are connected in sequence via revolute joints with NiTi rod pins (1 mm in diameter). Two additional NiTi rods, each 0.6 mm in diameter, function as the elastic backbones. The total mass of the manipulator is 8.4 g. For actuation, 6 nylon cables, each 0.5 mm in diameter, are used as driving tendons. Inside the actuation unit (Fig. 5B), each tendon is connected to a 3D-printed resin capstan, which is driven by a brushless motor (LKMTECH MF5015, with an integrated motor driver). The control algorithm was implemented in Python at 20 Hz on a 64-bit Windows 10 PC (i5-8265U), which communicates with the 6 motors via the RS-485 protocol. The model parameters are provided in Table 1, where SI units are adopted and omitted for brevity. These parameter settings were used as the default in the subsequent experiments, except in the section “Comparisons of trajectory frequency and model parameters”, where the values of γ, $\sigma_{p}$, and $\sigma_{\psi }$ were varied to compare performance.

**Table 1.** Model parameters in SI units

Parameter	Value
$L_{1}$, $L_{2}$, $L_{3}$	0.09, 0.09, 0.09
$r_{1}$, $r_{2}$, $r_{3}$	0.005, 0.0035, 0.002
α	1
β	0.5
γ	0.5
$\sigma_{p}$	0.35
$\sigma_{\psi }$	2.5
$\xi_{0}$	$0_{9\times 1}$
$P_{0}$	$0_{9\times 9}$
$Q_{k}$	$I_{9}$
$R_{k}$	$0.5{\left\|u_{k}-u_{k-1}\right\|}^{2}I_{3}$

To obtain the real-time pose feedback, 3 reflective markers, each 6 mm in diameter and with a combined weight of 0.6 g, are attached to the robot’s end-effector. An infrared webcam (640 × 480 resolution) with integrated infrared light-emitting diodes is positioned with its lens perpendicular to the manipulator’s bending plane, at a distance of 38.5 cm. The camera was calibrated using the MATLAB Computer Vision Toolbox. Fig. 5D shows an image captured by the infrared camera, where the reflective markers are clearly distinguishable from the background due to their high infrared reflectivity. Leveraging the markers’ high reflectivity and the manipulator’s planar motion, the end-effector pose can be efficiently tracked in real time at 30 Hz by identifying the positions of the 3 markers using the Python OpenCV library. The extracted end-effector pose is then sent to the main controller thread in real time via the User Datagram Protocol (UDP). Considering noise and disturbances, a 4th-order Butterworth low-pass filter with a cutoff frequency of 2 Hz was employed to preprocess the end-effector pose measurements and the actuation inputs used in the Jacobian estimation.

### Trajectory tracking

To validate the proposed online Jacobian error compensation method, 3 trajectory tracking experiments were conducted. For comparison, in addition to the proposed method, the continuum robot was also controlled to follow the trajectory in each experiment using only the Jacobian derived from the PCC model, without the online compensation.

**Fig. 6.**
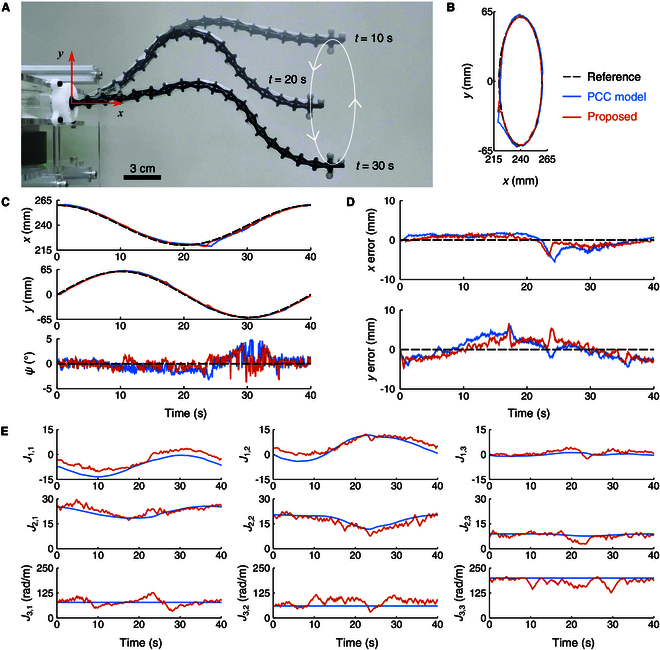
End-effector position trajectory tracking with a horizontal attitude (Traj. 1). (A) Composite image taken from the experiment. (B) Position trajectories. (C) Time plots of position and attitude. (D) Position tracking errors. (E) Jacobian elements.

In the first experiment (Traj. 1), the robot was instructed to follow an end-effector position trajectory while keeping a horizontal attitude, as depicted by the composite image in Fig. 6. The reference position trajectory was defined by the equations $x_{r}=240+20\;\text{cos}\left(\frac{2\pi }{T}t\right)$ mm and $y_{r}=60\;\text{sin}\left(\frac{2\pi }{T}t\right)$ mm, with the period *T* = 40 s. The measured trajectories and tracking errors (measurement minus reference) are presented in Fig. 6B to D. The root mean square errors (RMSEs) of the proposed method in the *x*, *y*, and ψ directions are 1.1 mm, 2.1 mm, and 1.1°, respectively, and the mean absolute errors (MAEs) are 0.9 mm, 1.8 mm, and 0.8°. Both metrics are lower than those of the PCC model without online compensation, which exhibits RMSEs of 1.6 mm, 2.3 mm, and 1.4° and MAEs of 1.4 mm, 1.9 mm, and 1.1° in the *x*, *y*, and ψ directions, as summarized in Table 2.

**Table 2.** Results for end-effector trajectory tracking

	Proposed (RMSE/MAE)			PCC model (RMSE/MAE)		
Experiment	*x*/mm	*y*/mm	ψ/(°)	*x*/mm	*y*/mm	ψ/(°)
Traj. 1	1.1/0.9	2.1/1.8	1.1/0.8	1.6/1.4	2.3/1.9	1.4/1.1
Traj. 2	0.6/0.4	0.8/0.6	1.5/1.3	1.3/1.0	0.9/0.6	2.1/1.9
Traj. 3	0.9/0.8	1.9/1.7	1.3/1.1	1.7/1.4	2.1/1.7	1.6/1.4

**Fig. 7.**
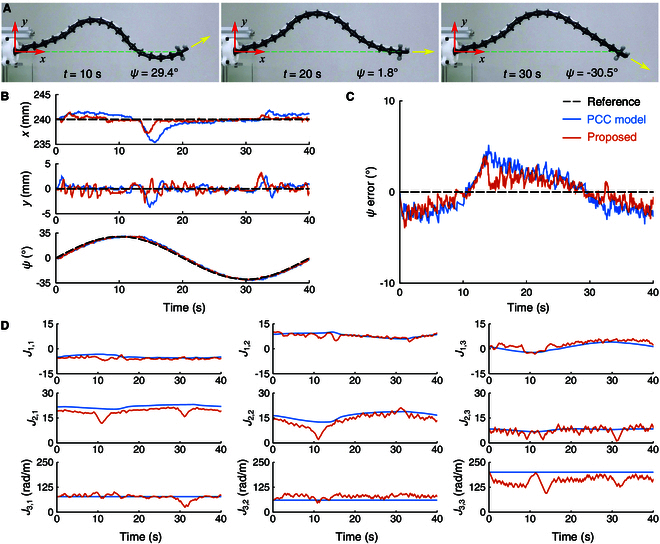
End-effector attitude trajectory tracking, with the position controlled to remain stationary (Traj. 2). (A) Sequential images taken from the experiment. (B) Time plots of position and attitude. (C) Attitude tracking errors. (D) Jacobian elements.

In the second experiment (Traj. 2), the continuum manipulator demonstrated its ability to regulate the end-effector attitude as shown in Fig. 7. The reference attitude was specified as $\psi_{r}=30^{\circ }\text{sin}\left(\frac{2\pi }{T}t\right)$ with the same period *T* = 40 s, while the robot was controlled to keep the end-effector position stationary. Fig. 7A illustrates a sequence of images showing the robot’s configuration at different end-effector attitudes, along with the trajectory measurements (Fig. 7B) and tracking errors (Fig. 7C). As listed in Table 2, the proposed method achieves RMSEs of 0.6 mm, 0.8 mm, and 1.5°, and MAEs of 0.4 mm, 0.6 mm, and 1.3° in the *x*, *y*, and ψ directions, respectively, outperforming the PCC model, which has RMSEs of 1.3 mm, 0.9 mm, and 2.1°, and MAEs of 1.0 mm, 0.6 mm, and 1.9°.

**Fig. 8.**
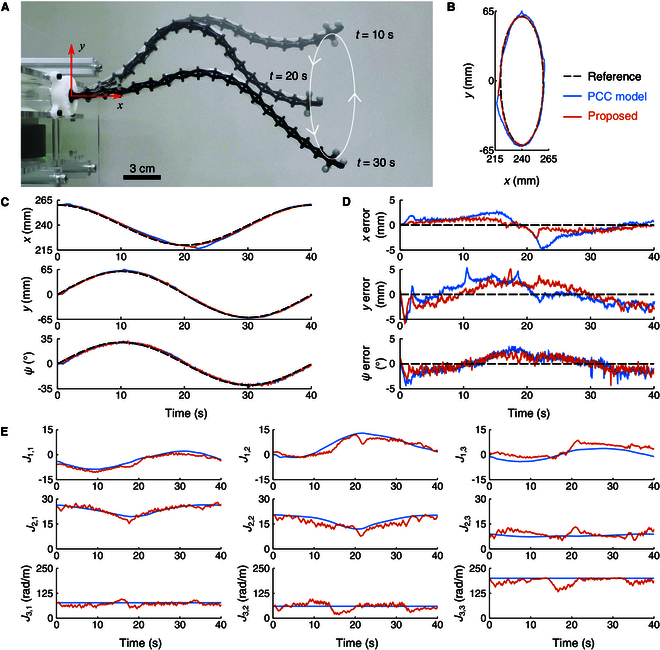
Hybrid tracking of end-effector position and attitude trajectories (Traj. 3). (A) Composite image showing the tracking process. (B) Position trajectories. (C) Time plots of position and attitude. (D) Tracking errors. (E) Jacobian elements.

In the third experiment (Traj. 3), the robot was required to simultaneously follow both the position and attitude trajectories of the end-effector, as shown in Fig. 8. The position trajectory from the first experiment and the attitude trajectory from the second experiment were employed as references. The measurement results are shown in Fig. 8B to D. The proposed method yields RMSEs of 0.9 mm, 1.9 mm, and 1.3°, along with MAEs of 0.8 mm, 1.7 mm, and 1.1° in the *x*, *y*, and ψ directions, respectively. These results demonstrate better performance than the PCC model, which shows RMSEs of 1.7 mm, 2.1 mm, and 1.6°, and MAEs of 1.4 mm, 1.7 mm, and 1.4°, as listed in Table 2.

Figs. 6E, 7E, and 8E illustrate the Jacobian elements of the proposed method and the PCC model for the 3 tracking experiments, respectively. Additionally, the Jacobian errors estimated by the proposed method during the 3 trajectories are presented in Figs. S1 to S3. Compared to the PCC model, the proposed method compensated for the robot Jacobian online using real-time measurement data, resulting in lower tracking errors.

In summary, the results from the 3 trajectory tracking experiments demonstrate that the proposed online Jacobian error compensation method improves tracking accuracy in both position and attitude.

### Comparisons of trajectory frequency and model parameters

This section demonstrates the performance of the proposed method under variations in the trajectory period *T* and the model parameters γ, $\sigma_{p}$, and $\sigma_{\psi }$. For evaluation, the robot was instructed to execute Traj. 3 ($x_{r}=240+20\;\text{cos}\left(\frac{2\pi }{T}t\right)$ mm, $y_{r}=60\;\text{sin}\left(\frac{2\pi }{T}t\right)$ mm, and $\psi_{r}=30^{\circ }\text{sin}\left(\frac{2\pi }{T}t\right)$, as described previously) under different parameter settings. The tracking result of Traj. 3 from the previous section, with *T* = 40 s, *γ* = 0.5, $\sigma_{p}=0.35$, and $\sigma_{\psi }=2.5$, is used as the baseline and illustrated again in Table 3 for comparison.

**Table 3.** Results for varying parameters in trajectory tracking

	End-effector error (RMSE/MAE)		
Parameter	*x*/mm	*y*/mm	ψ/(°)
Baseline	0.9/0.8	1.9/1.7	1.3/1.1
*T* = 20 s	3.1/2.4	3.6/3.0	2.8/2.4
*T* = 10 s	6.9/5.1	6.5/5.3	4.8/4.3
*T* = 5 s	11.3/8.7	15.6/12.7	8.4/7.4
γ = 1	2.4/1.9	4.1/3.1	2.4/1.9
γ = 0	1.5/1.3	1.8/1.5	1.5/1.2
$\sigma_{p}=1.4,\sigma_{\psi }=10$	1.4/1.2	2.3/1.9	1.6/1.3
$\sigma_{p}=0.0875,\sigma_{\psi }=0.625$	2.0/1.5	2.1/1.8	2.2/1.9

**Fig. 9.**
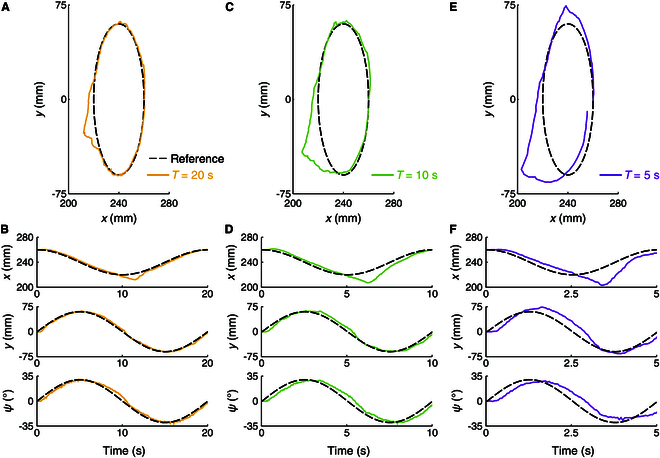
Comparison of tracking trajectories with different periods. Position trajectories and time plots of position and attitude are shown for (A and B) *T* = 20 s, (C and D) *T* = 10 s, and (E and F) *T* = 5 s.

First, the continuum manipulator was commanded to follow trajectories with varying periods: *T* = 20 s, 10 s, and 5 s. The results are presented in Fig. 9, with the tracking errors (RMSEs and MAEs) listed in Table 3. As *T* decreases, the tracking errors increase significantly. This behavior is attributed to the quasi-static nature of the proposed method, which assumes slow robot movement and ignores dynamic effects. Shorter trajectory periods challenge this assumption, leading to reduced tracking accuracy. In practice, the trajectory frequency should be kept low to achieve high tracking precision, which is a limitation of the proposed method.

**Fig. 10.**
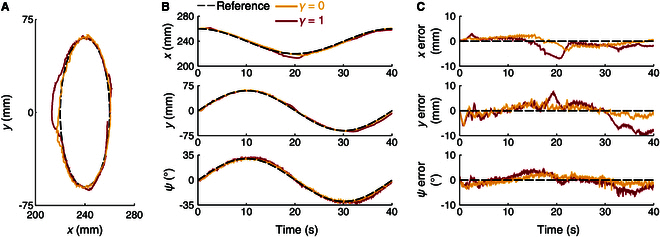
Comparison of *γ* in trajectory tracking. (A) Position trajectories. (B) Time plots of position and attitude. (C) Tracking errors.

Then, the effect of *γ*, the fading rate that governs the influence of the previous estimation on the current Jacobian error estimate, was investigated. Two tracking experiments were conducted with *γ* = 0 (the minimum value) and *γ* = 1 (the maximum value), respectively. The results are shown in Fig. 10, with the corresponding tracking errors listed in Table 3. For *γ* = 0, the Jacobian estimation did not rely on any historical information. Compared to the baseline, the tracking error is slightly lower in the *y* direction but higher in both the *x* and ψ directions. In contrast, when *γ* = 1, the tracking errors are significantly larger because the estimated Jacobian became overly reliant on previous estimations, leading to poor performance. The results indicate that *γ* should be tuned within [0,1] to achieve a balance between the current and historical estimations, ensuring accurate tracking performance.

**Fig. 11.**
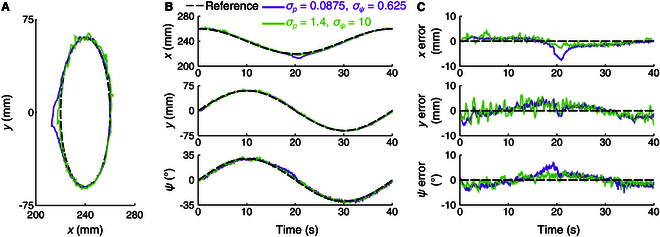
Comparison of $\sigma_{p}$ and $\sigma_{\psi }$ in trajectory tracking. (A) Position trajectories. (B) Time plots of position and attitude. (C) Tracking errors.

Finally, the impact of thresholds $\sigma_{p}$ and $\sigma_{\psi }$, which regulate the update magnitudes of the estimated Jacobian, was examined. Two tracking experiments were conducted: one with $\sigma_{p}=0.0825$ and $\sigma_{\psi }=0.625$ (one-fourth of the baseline) and another with $\sigma_{p}=1.4$ and $\sigma_{\psi }=10$ (4 times the baseline). The results are shown in Fig. 11, with the corresponding tracking errors summarized in Table 3. For the smaller thresholds ($\sigma_{p}=0.0825$ and $\sigma_{\psi }=0.625$), the trajectory exhibited greater deviation from the reference due to insufficient updates in the Jacobian estimation. In contrast, when $\sigma_{p}=1.4$ and $\sigma_{\psi }=10$, the trajectory deviated less but experienced noisy oscillations caused by excessive variation of the estimated Jacobian. These findings suggest that moderate $\sigma_{p}$ and $\sigma_{\psi }$ should be adopted to reduce tracking deviation and oscillation.

### Performance under external disturbances

This section evaluates the performance of the proposed method when encountering external disturbances. Two experiments were conducted by hanging unexpected payloads on the continuum manipulator.

**Fig. 12.**
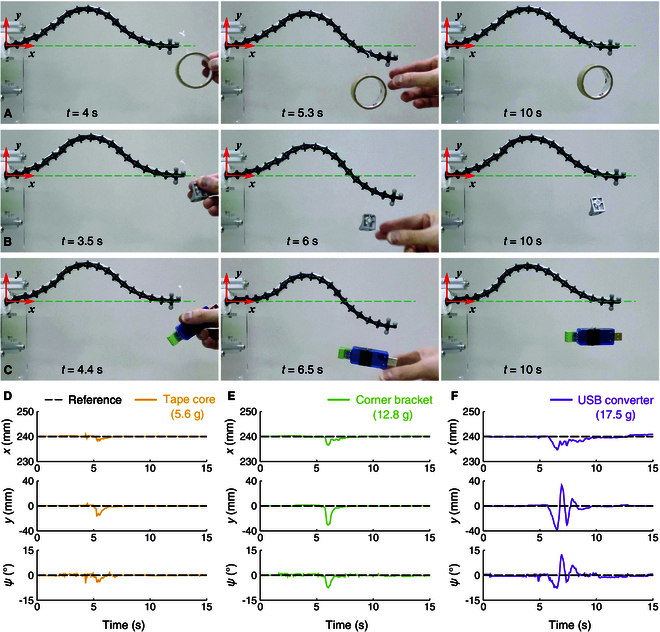
Adding disturbances by hanging payloads while maintaining the desired pose stationary. Sequential images show hanging (A) a tape core, (B) a corner bracket, and (C) a USB converter. (D to F) Time plots of end-effector position and attitude under different payloads.

In the first experiment, the robot was initially controlled to maintain a stationary end-effector pose. To apply the disturbance, a payload was hung on the robot using a nylon cable. This experiment was repeated 3 times with objects of different weights, as shown in Fig. 12A (a tape core, 5.6 g), Fig. 12B (a corner bracket, 12.8 g), and Fig. 12C (a USB converter, 17.5 g). After the payload was applied, the robot deviated temporarily from its original configuration but quickly returned to the desired pose, as illustrated in Fig. 12D to F, with larger deviations occurring for heavier payloads, as expected. The largest deviation happened when the USB converter was suspended, which is twice the weight of the continuum manipulator. In this situation, the maximum absolute errors (MaxAEs) of the end-effector in the *x*, *y*, and ψ directions are 5.3 mm, 38.9 mm, and 12.5°, respectively, as listed in Table 4.

**Table 4.** Results for adding disturbances in a stationary pose

	End-effector MaxAE		
Payload	*x*/mm	*y*/mm	ψ/(°)
Tape core (5.6 g)	1.6	16.0	4.8
Corner bracket (12.8 g)	3.4	30.5	7.4
USB converter (17.5 g)	5.3	38.9	12.5

**Fig. 13.**
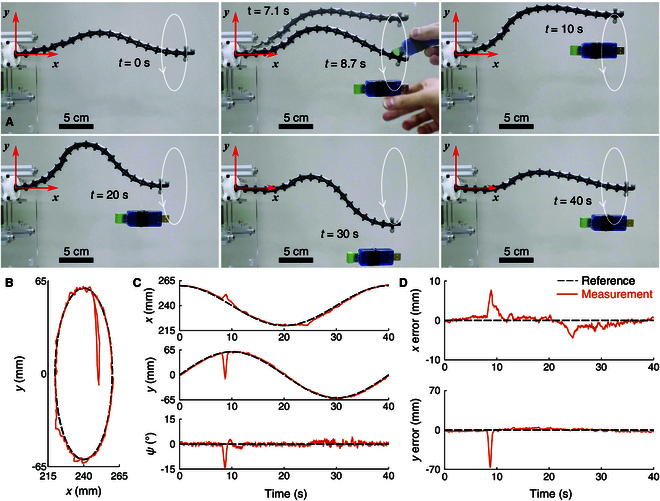
Adding disturbance during end-effector trajectory tracking. (A) Sequence of images illustrating the robot executing the trajectory under an unexpected disturbance from a USB converter. (B) Position trajectories. (C) Time plots of position and attitude. (D) Position tracking errors.

In the second experiment, the USB converter was added as an unexpected disturbance while the robot was executing an end-effector position trajectory with a horizontal attitude, as shown in Fig. 13A. The robot was commanded to follow the same Traj. 1 described in the section “Trajectory tracking”. At *t* = 8 s, the USB converter was suspended, causing the robot to temporarily deviate from the reference trajectory. The robot recovered quickly and returned to follow the trajectory stably as demonstrated in Fig. 13B to D, with MaxAEs of the end-effector in the *x*, *y*, and ψ directions as 7.7 mm, 66.8 mm, and 14.1°, respectively. After the payload was applied (*t* = 8 to 40 s), the trajectory tracking RMSEs are 1.7 mm, 7.6 mm, and 1.9°, and the MAEs are 1.2 mm, 3.0 mm, and 1.0° in the *x*, *y*, and ψ directions, respectively, which are summarized in Table 5.

**Table 5.** Results for adding disturbance (a USB converter, 17.5 g) during trajectory tracking

	End-effector error		
Metric	*x*/mm	*y*/mm	ψ/(°)
RMSE	1.7	7.6	1.9
MAE	1.2	3.0	1.0
MaxAE	7.7	66.8	14.1

The experimental results demonstrate the ability of the continuum robot to handle unexpected disturbances, showcasing the versatility and robustness of the proposed method.

## Conclusion

In this work, a hybrid model-based and data-driven method is proposed for controlling a continuum robot. By utilizing real-time actuation inputs and end-effector pose measurements, the model-based Jacobian of the robot is updated online through a Kalman filter to account for modeling inaccuracies. This approach eliminates the need for offline dataset collection and training. Experiments conducted on a planar continuum manipulator demonstrate the effectiveness of the proposed method in improving tracking accuracy for both position and attitude. Additionally, the effects of various model parameters are analyzed through comparative experiments. The robustness of the proposed method against external disturbances is also validated. However, due to the neglect of robot dynamics, the proposed method is currently limited to low operation velocities. Future work will focus on extending the method to incorporate the dynamic effects of continuum robots. Furthermore, we aim to advance this hybrid model-based and data-driven framework to enhance the accuracy and efficiency of onboard state estimation for continuum robots.

## Data Availability

The data that support the findings of this study are available from the corresponding author upon reasonable request.
