# Supplementary material for: Model-Based Control of a Continuum Manipulator with Online Jacobian Error Compensation Using Kalman Filtering
Source: Cyborg Bionic Syst. 2025 Aug 7;6:0339. doi: 10.34133/cbsystems.0339 (PMC12329213; doi:10.34133/cbsystems.0339)
Supplement: Supplementary 1 — Figs. S1 to S3 Movie S1 [file cbsystems.0339.f1.zip › supplementary.pdf]

Supplementary Materials for

Model-Based Control of a Continuum Manipulator with Online Jacobian Error Compensation Using Kalman Filtering

Yujia Zhai[1], Jihao Xu[1], Hangjie Mo[2], Chunqi Zhang[1], and Dong Sun[1,*]

[1]Department of Biomedical Engineering, City University of Hong Kong,
Tat Chee Avenue, Hong Kong SAR, China.
[2]School of Management, Hefei University of Technology, Hefei 230009, China.

[*]Address correspondence to: medsun@cityu.edu.hk

This file includes:

Figs. S1 to S3

Other Supplementary Material for this manuscript includes the following:

Movie S1

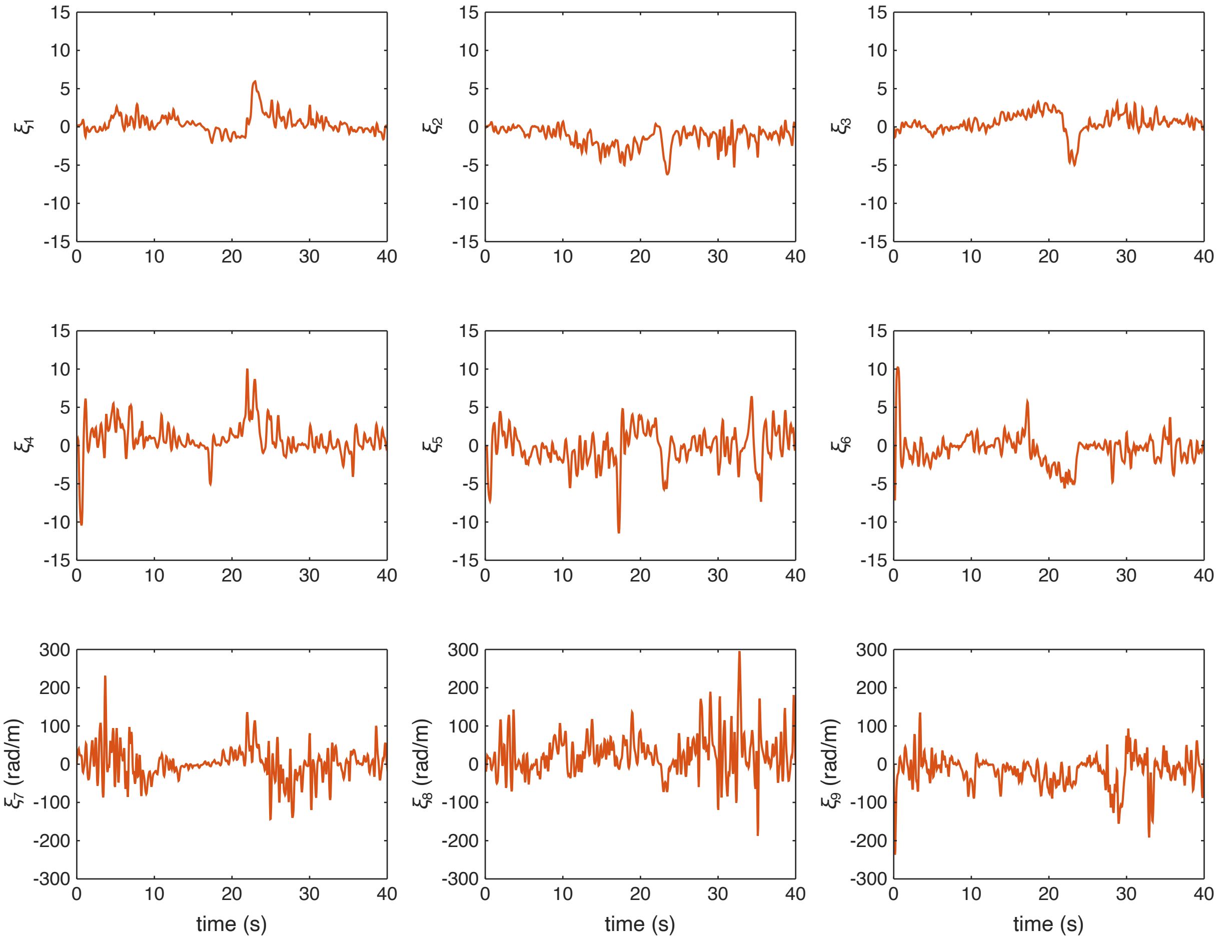

Fig. S1. Elements of the estimated Jacobian error during the tracking of Traj. 1.

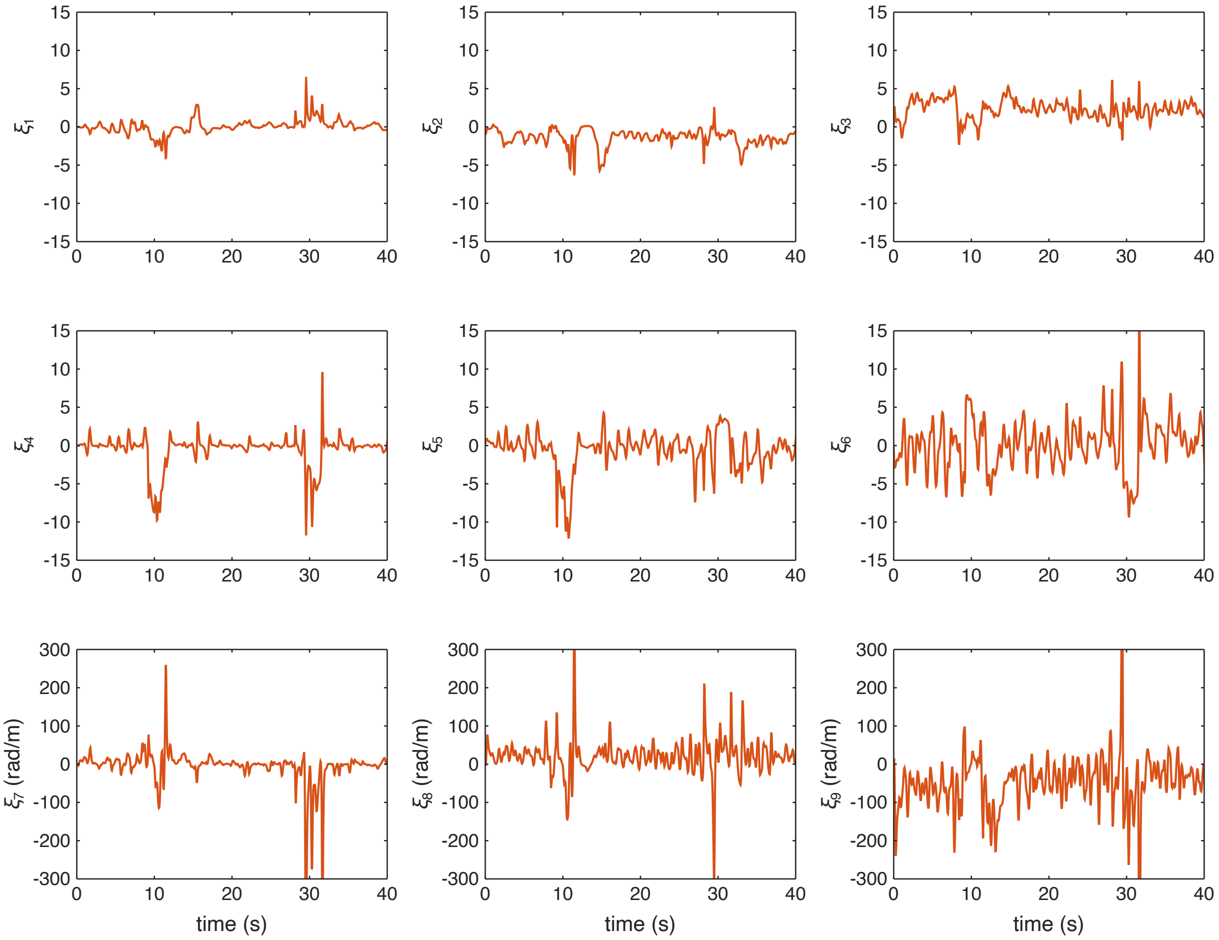

Fig. S2. Elements of the estimated Jacobian error during the tracking of Traj. 2.

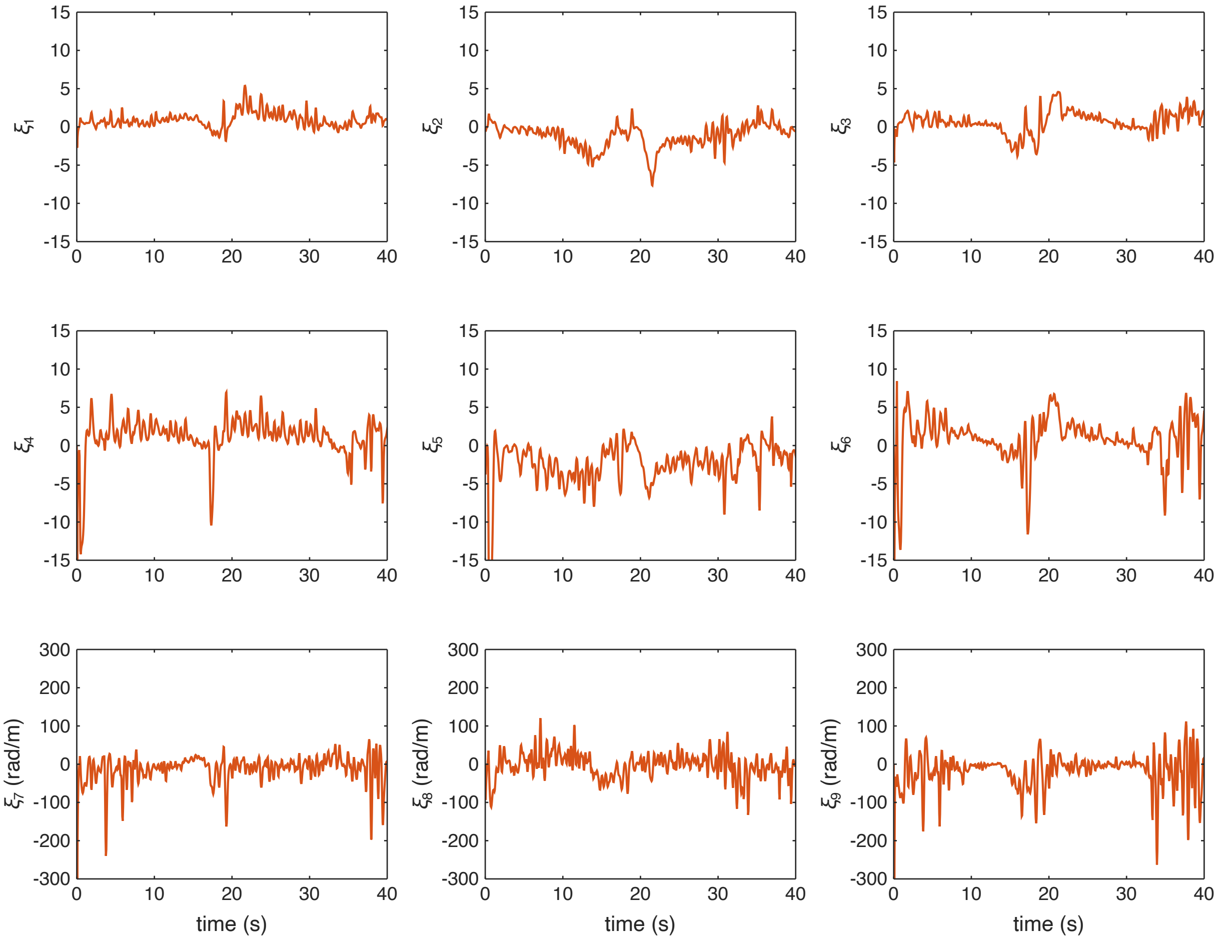

Fig. S3. Elements of the estimated Jacobian error during the tracking of Traj. 3.
